# Assessment of the Impact of the 2003 and 2006 Heat Waves on Cattle Mortality in France

**DOI:** 10.1371/journal.pone.0093176

**Published:** 2014-03-25

**Authors:** Eric Morignat, Jean-Baptiste Perrin, Emilie Gay, Jean-Luc Vinard, Didier Calavas, Viviane Hénaux

**Affiliations:** 1 Agence nationale de sécurité sanitaire de l'alimentation, de l'environnement et du travail (Anses), Unité Epidémiologie du Laboratoire de Lyon, Lyon, France; 2 Direction Générale de l'Alimentation (DGAl), Bureau de la santé animale, Paris, France; Auburn University, United States of America

## Abstract

**Objectives:**

While several studies have highlighted and quantified human mortality during the major heat waves that struck Western Europe in 2003 and 2006, the impact on farm animals has been overlooked. The aim of this study was to assess the effect of these two events on cattle mortality in France, one of the most severely impacted countries.

**Methods:**

Poisson regressions were used to model the national baseline for cattle mortality between 2004 and 2005 and predict the weekly number of expected deaths in 2003 and 2006 for the whole cattle population and by subpopulation based on age and type of production. Observed and estimated values were compared to identify and quantify excess mortality. The same approach was used at a departmental scale (a French department being an administrative and territorial division) to assess the spatio-temporal evolution of the mortality pattern.

**Results:**

Overall, the models estimated relative excess mortality of 24% [95% confidence interval: 22–25%] for the two-week heat wave of 2003, and 12% [11–14%] for the three-week heat wave of 2006. In 2003, most cattle subpopulations were impacted during the heat wave and some in the following weeks too. In 2006, cattle subpopulations were impacted for a limited time only, with no excess mortality at the beginning or after the heat wave. No marked differences in cattle mortality were found among the different subpopulations by age and type of production. The implications of these results for risk prevention are discussed.

## Introduction

Definitions of heat wave vary, but these extreme events are widely recognized as a sustained period of excessively hot weather compared to mean temperatures for that area and season. More precisely, the World Meteorological Organization—along with other meteorological organizations—defines a heat wave as a period of more than five consecutive days with a daily maximum temperature exceeding by 5°C the average maximum temperature of the reference period (1961–1990) [1]. In the summers of 2003 and 2006, France and most of Western Europe experienced the two most severe heat waves since 1950. From August 1^st^ to 5^th^ 2003, the average maximum temperature in France increased from 25°C—a value close to the seasonal mean— to 37°C, where it remained until August 16^th^ before dropping again to 28°C. Almost all metropolitan France was exposed to the heat wave, with temperatures exceeding 35°C for at least nine days in 61 of the 96 French departments [2] (a department being a French administrative and territorial division covering a mean surface area of 5,800 km^2^). The heat wave of 2006 was also particularly severe. Although temperatures did not reach the levels recorded in 2003, they steadily increased from mid-June onwards, exceeding 30°C between July 10^th^ and 28^th^. Temperatures in most of the country began to drop on July 29^th^, but remained high until early August in the South East. The length of this heat wave contributed to making July 2006 the hottest in France since 1950 [3].

The duration, intensity, and geographic extent of the 2003 heat wave caused a major rise in human mortality [4]–[7] and substantial economic losses, prompting the implementation of preventive measures which limited the human health impact in 2006 [7]. High temperatures have also been shown to impair animal physiology, metabolism and general health [8]–[10], leading to poor animal welfare and financial losses [11]. Although cattle renderers detected and notified *a posteriori* excess mortality in farm animals—especially cattle in 2003—the specific impact of these two heat waves on animal mortality has never been explicitly evaluated.

The goal of this study was to assess the impact of the heat waves of August 2003 and July 2006 on cattle mortality on both a national and departmental scale. Total mortality associated with a meteorological or health event can be evaluated through the retrospective modeling of mortality fluctuations. This approach is commonly implemented by human health agencies [5], [12], [13], and has recently been used to evaluate the impact of the Bluetongue outbreak among cattle in France [14].

## Material and Methods

### Mortality data

Mortality data were extracted from the French National Cattle Register, managed by the French Ministry of Agriculture. This register gathers information about cattle herds (identification number, location) and animal movements (date and nature of the movement, e.g. entry/exit; reason of the movement, e.g. birth/purchase for entries, death/slaughter/sale for exits). This database was used to determine the daily number of living or dead cattle at a herd scale. The analysis focused on unplanned farm deaths or euthanasia between January 1^st^ 2003 and December 31^st^ 2006 in mainland France, except for the greater Paris region where there were too few cattle. In all, about 45 million cattle were registered and 4.9 million died from unforeseen causes during the 2003–2006 period.

Each animal was attributed a type of production (dairy or beef cattle) according to its breed and classified by age (0 to 7 days, 8 days to 6 months, 6 months to 2 years, 2 to 5 years, and over 5 years). The numbers of dead and live cattle were computed per department, production type and age class by aggregating daily data to obtain weekly figures, as the date of death was not very reliable. Indeed, preliminary data analyses suggested that the date reported by farmers is more likely to correspond to the date of carcass collection rather than the actual date of death. Since no carcasses are picked up on weekends or bank holidays, there can be a difference of one to two days between the date of death and the collection date. Data were managed with the Toad for MySQL development tool.

### Statistical analyses

The baseline mortality rate was defined as λ_t_ = m_t_/N_t_ where m_t_ is the number of observed deaths over week t and N_t_ the number of animal weeks at risk of dying. Nt was computed by summing the number of days each animal was present during week t then dividing this total by seven. Given this definition, one animal week can represent one animal present on seven days or seven on one day, for example. Seasonal fluctuations of this baseline mortality rate were modeled over a two-year calibration period (2004–2005 inclusive) using a time-dependent Poisson regression with an overdispersion parameter. We took overdispersion into consideration since it is frequently encountered with count data when unidentified factors or clustering are not taken into account in the model. The hierarchical structure of the data (cattle grouped into herds belonging to an administrative and territorial division) was not considered because models including random effects cannot make predictions outside the population used to calibrate the model. Besides, although data were collected between 2002 and 2008, the model was calibrated on data from 2004–2005 only for two reasons: first, because information collected the first year (training period) was not fully reliable, and second, the Bluetongue outbreak in 2007 and 2008 considerably changed the mortality pattern [14].

Three different models were used to evaluate variations in mortality rates for different spatial scales and subpopulations.

#### National general model

This first model estimated the mean number of dead cattle per week (μ_t_) countrywide for the whole cattle population:
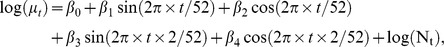
where the trigonometric functions describe annual and semestrial seasonal variations in cattle mortality [14], with t ranging from 1 (first week of 2004) to 105 (last week of 2005). The number of cattle at risk (N_t_) was included in the model as an offset.

#### National categorical model

The previous model was extended to include the type of production and age as covariates, the objective being to investigate variations in mortality rates by subpopulation. It can be written in a concise format as follows according to the Wilkinson-Rogers notation:
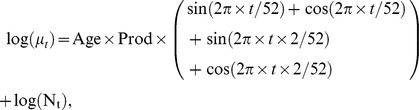
where Age and Prod denote variables representing the age class and production type respectively. Interactions between time and the covariates were introduced to reveal different temporal patterns between cattle subpopulations.

#### Departmental categorical models

Spatio-temporal variations in mortality were investigated by fitting the categorical model to each department. Five departments in southern France (Bouches du Rhône, Gard, Hérault, Vaucluse and Var) were excluded as the models lacked convergence because there were too few animals in some subpopulations. Therefore, mean mortality rates by cattle subpopulation were evaluated in 81 departments.

To reduce the influence of unexplained aberrations in mortality during the calibration period, each model was run twice so as to weight observations by the inverse of the squared standardized Anscombe residuals from the initial model [15]. This approach ensures that values with high residuals are given lower weights in the regression. In all the models, the weekly expected number of dead cattle (E) was extrapolated for 2003 and 2006, with 99% prediction intervals computed according to the method proposed by Farrington [15].

For the two national models, the difference between observed (O) and expected (E) numbers of dead cattle, rounded up to the nearest integer, was computed for each week of 2003 and 2006. A positive difference indicated excess mortality, while a negative difference denoted a mortality deficit. Relative excess mortality was defined as (O-E)/E. In order to compare subpopulations, standardized mortality ratios (SMRs) were calculated on the basis of the ratio of observed to expected numbers of dead cattle (O/E), with the related confidence intervals. Exact confidence intervals [16] were approximated by applying the Chi-square approximation proposed by Wilson and Hilferty [17]. Given the heterogeneity of population density in the different departments, only SMRs (and associated confidence intervals) were computed for the departmental models. Statistical analyses were performed with the R software [18].

While it would be ideal to analyze the correlation between weekly temperatures and SMRs at department level, daily temperature data were available for a limited number of weather stations and did not allow us to estimate a mean weekly temperature for every department. Météo France, the French national meteorological service (http://france.meteofrance.com/france/actu/bilan/archives/2006/canicule?page_id=10043), has generated maps showing the distribution of the number of days with temperatures above 35°C (referred to hereafter as “hot days”) during the heat waves of 2003 and 2006. This spatial pattern was used as an indicator of the heat waves' magnitude and duration on a local scale.

## Results

In 2003, the heat wave started on August 1^st^ and finished on August 15^th^, including week 32 (August 4–10) and five days of week 33 (August 11–17). In 2006, the heat wave started on July 10^th^ and ended on July 28^th^, including week 28 (July 10–16), week 29 (July 17–23) and five days of week 30 (July 24–30).

### Model validation

**Figure 1:**
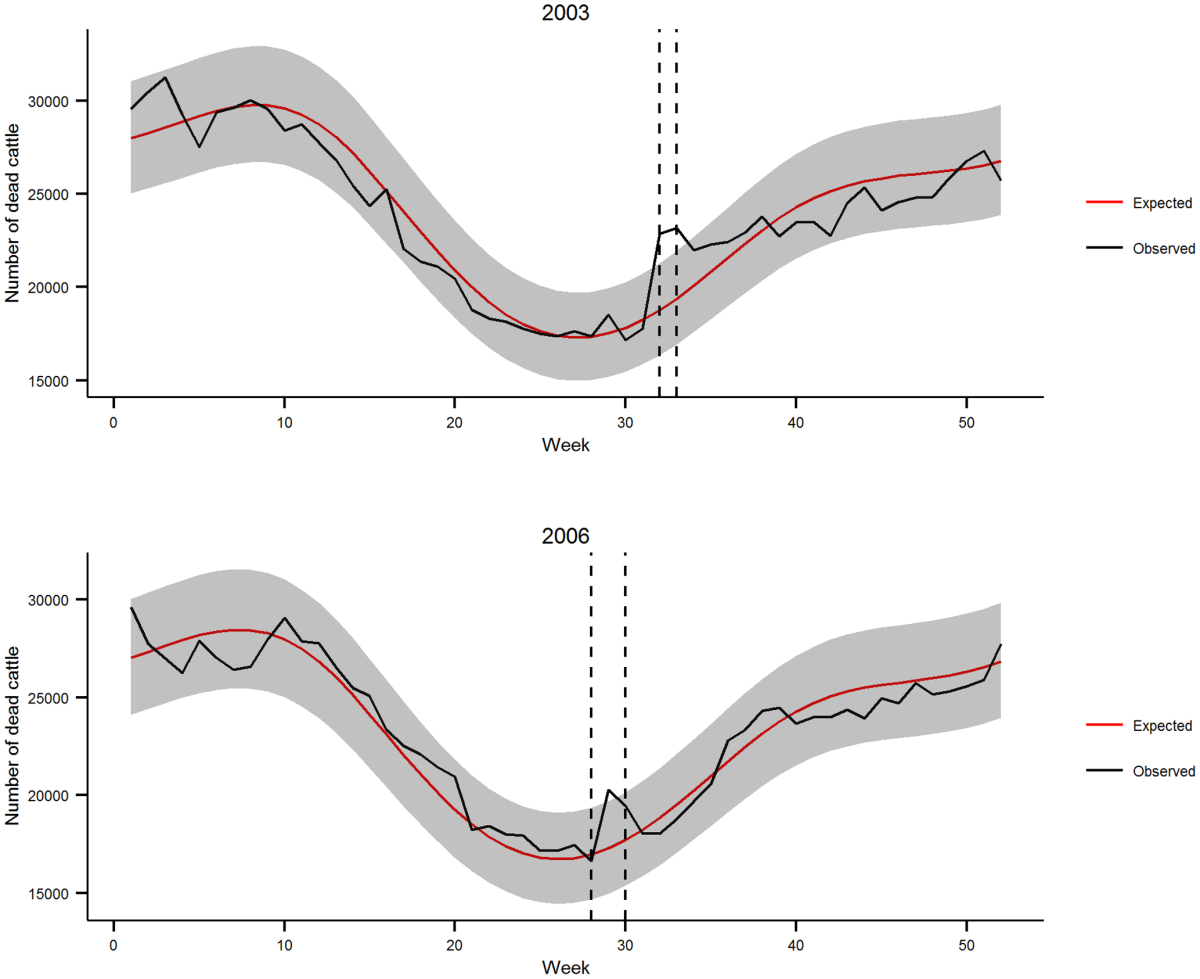
Overall weekly cattle mortality in France in 2003 and 2006. Dashed lines indicate the heat wave period in 2003 (weeks 32–33) and 2006 (weeks 29–30). Expected mortality is represented by the red line (with the 99% confidence interval in gray), and observed mortality by the dark line.

Model fit was evaluated by comparing predicted (E) and observed (O) numbers of dead cattle in 2003 and 2006 for the weeks outside the heat wave. The national general model provided a good fit to the baseline weekly mortality rate in both 2003 and 2006, with all observed mortality values outside the heat wave periods lying within the 99% prediction interval (Figure 1). Estimates and the corresponding standard errors of the parameters from the national general model are displayed in table 1.

**Table 1:** Estimates and standard errors of the coefficients from the national general model.

Coefficient	Estimate	Standard error
*β*_0_	−8.693	0.002
*β*_1_	0.067	0.003
*β*_2_	0.230	0.004
*β*_3_	0.004	0.003
*β*_4_	−0.081	0.004

**Figure 2:**
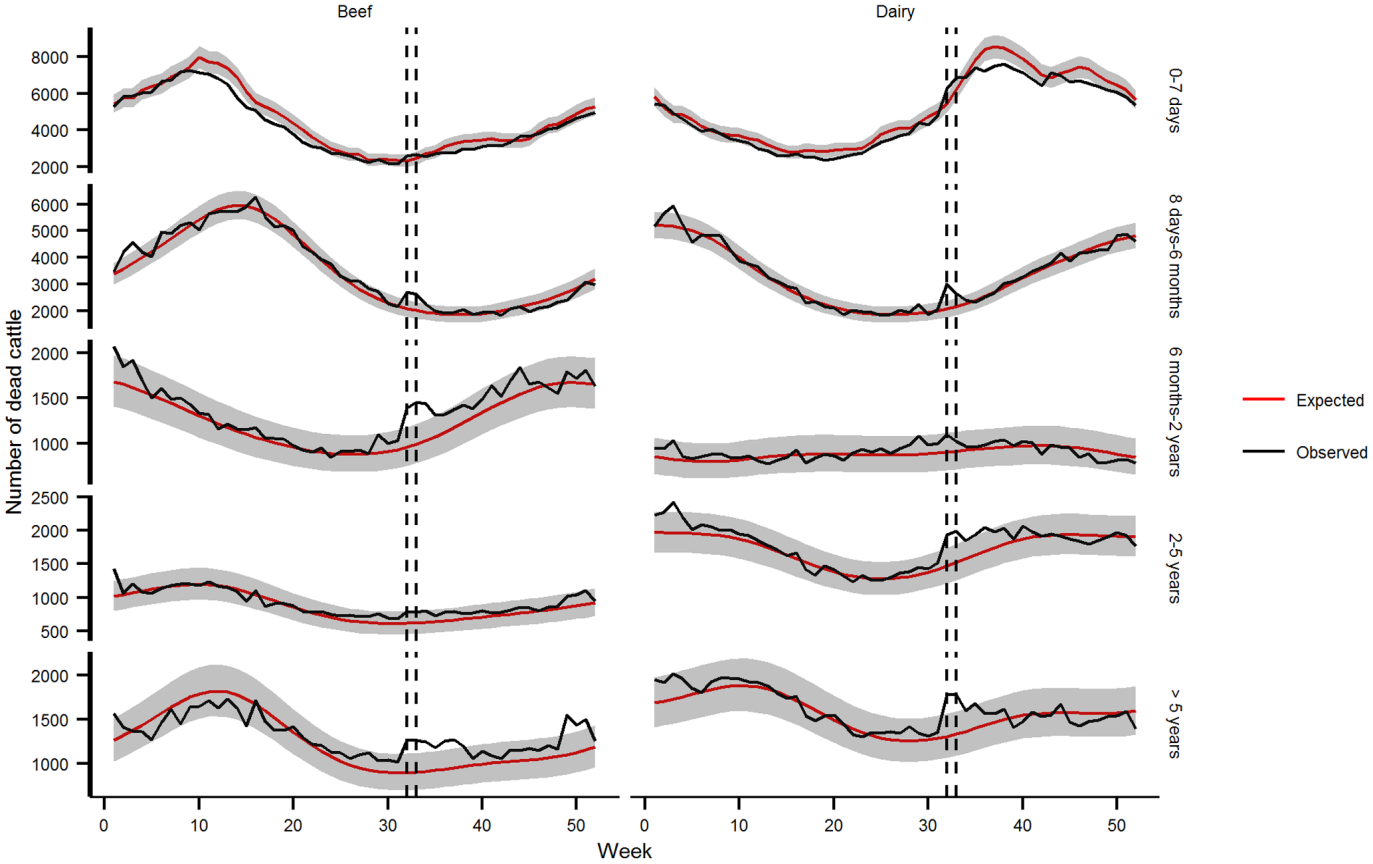
Comparison of observed and expected weekly cattle mortality in France in 2003 by age and production type. Dashed lines indicate the heat wave period. Expected mortality is represented by the red line (with the 99% confidence interval in gray), and observed mortality by the dark line.

**Figure 3:**
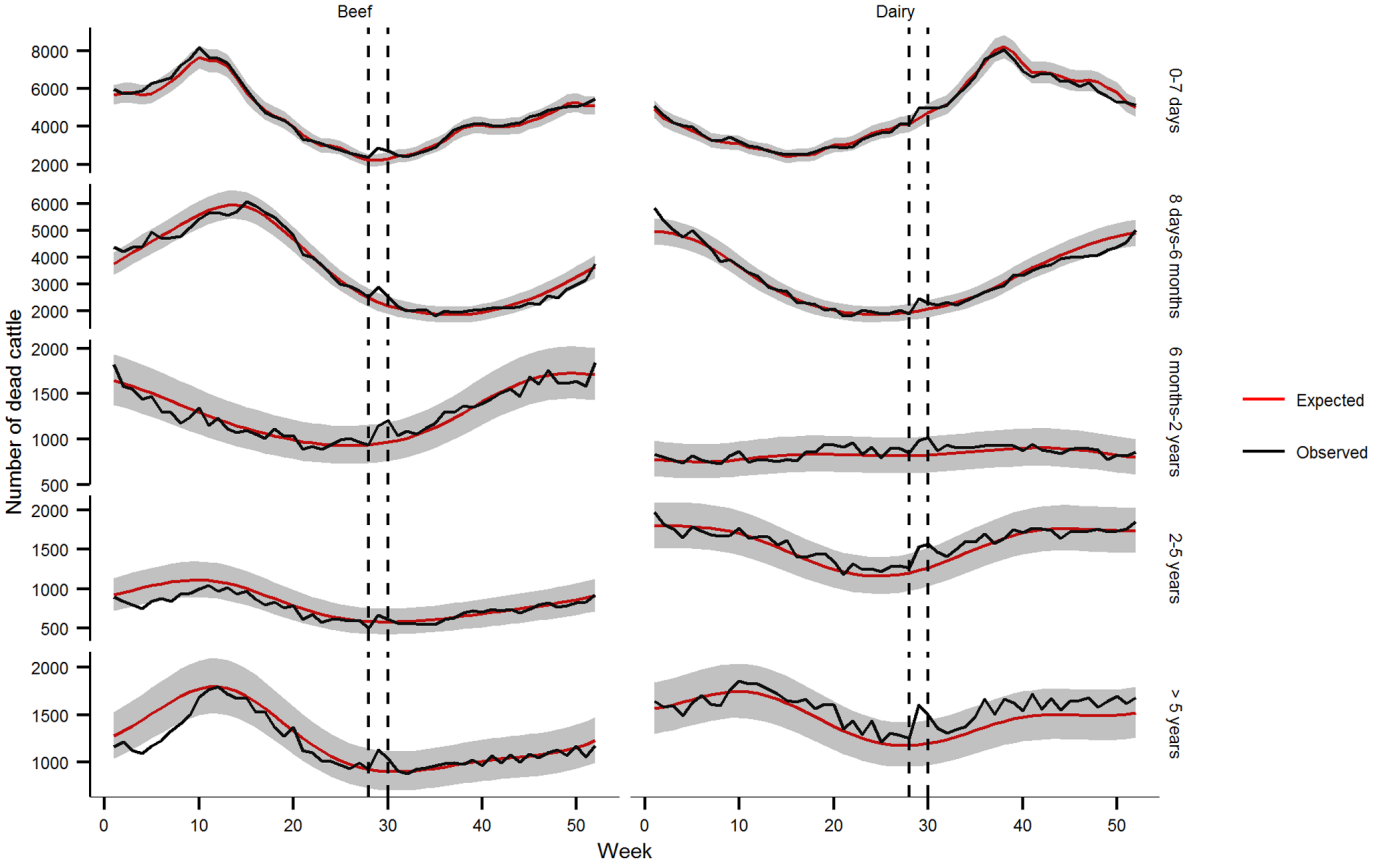
Comparison of observed and expected weekly cattle mortality in France in 2006 by age and production type. Dashed lines indicate the heat wave period. Expected mortality is represented by the red line (with the 99% confidence interval in gray), and observed mortality by the dark line.

The fit of the national categorical model was relatively good in 2003 for all subpopulations. On average, 3.1% [95% confidence interval: 1.7–5.4] of the observed weekly mortality values lay outside the 99% prediction interval. However, for calves 0 to 7 days old, observed mortality values were under the lower bound of the 99% prediction interval for 15 consecutive weeks in beef calves and 20 weeks in dairy calves (Figure 2). In 2006, on average 2.1% [1.0–4.0] of observed mortality values lay outside the 99% prediction interval (Figure 3), except for beef cattle over 5 years old, which showed a significant mortality deficit for six consecutive weeks in January-February 2006.

The fit of the departmental models varied. On average, 4.9% [4.3–5.7] of the departments exhibited excess weekly mortality and 7.6% [6.8–8.4] a mortality deficit in 2003. In 2006, 3.8% [3.3–4.5] of the departments had excess mortality and 3.9 % [3.3–4.5], a deficit.

### Impact of the 2003 heat wave on cattle mortality

#### National analysis

Significant excess mortality was found in weeks 32 and 33 in 2003, with 4,752 and 4,012 additional deaths corresponding to a relative excess of 26% and 21% respectively. Overall, 8,764 extra deaths were predicted to occur over the heat wave period, corresponding to relative excess mortality of 24.0% [22.0–25.0].

All cattle subpopulations showed excess mortality, which ranged from 27 to 47% during weeks 32 and 33, except for all calves 0 to7 days old and dairy cattle between 6 months and 2 years, whose relative excess mortality varied between 10 and 17% only. Excess mortality remained significantly high after the heat wave in four subpopulations: for 2 weeks in beef cattle between 6 months and 2 years, for 5 weeks in beef cattle over 5 years, for 3 weeks in dairy cattle between 2 and 5 years, and for 1 week in dairy cattle over 5 years (Figure 2). Overall, similar excess mortality rates were found for both types of production within each age class, except for animals aged between 6 months and 2 years. In this age group, beef cattle exhibited an SMR of 1.47 [1.40–1.54] versus 1.15 [1.10–1.20] for dairy cattle (Table 2).

**Table 2:** Cumulated excess mortality by cattle subpopulation during the 2003 and 2006 heat waves in France.

Subpopulation	2003^(1)^	2006^(2)^
	O/E^(3)^ 99% Confidence interval	O/E 99% Confidence interval
Beef 0–7 days	1.10 1.06 – 1.14	1.18 1.14 – 1.21
Dairy 0–7 days	1.13 1.10 – 1.15	1.06 1.04 – 1.08
Beef 8 days-6 months	1.30 1.26 – 1.35	1.13 1.10 – 1.17
Dairy 8 days-6 months	1.34 1.29 – 1.39	1.10 1.07 – 1.14
Beef 6 months-2 years	1.47 1.40 – 1.54	1.15 1.10 – 1.20
Dairy 6 months-2 years	1.17 1.10 – 1.23	1.16 1.11 – 1.22
Beef 2–5 years	1.27 1.19 – 1.35	1.02 0.96 – 1.09
Dairy 2–5 years	1.31 1.26 – 1.37	1.18 1.13 – 1.22
Beef > 5 years	1.40 1.33 – 1.47	1.13 1.08 – 1.18
Dairy > 5 years	1.35 1.29 – 1.41	1.22 1.18 – 1.27
Total	1.24 1.22 – 1.25	1.12 1.11 – 1.14

1 weeks 32 and 33 in 2003.

2 weeks 28, 29 and 30 in 2006.

3 O/E  =  SMR, O  =  observed mortality, E  =  expected mortality.

#### Departmental analysis

**Figure 4:**
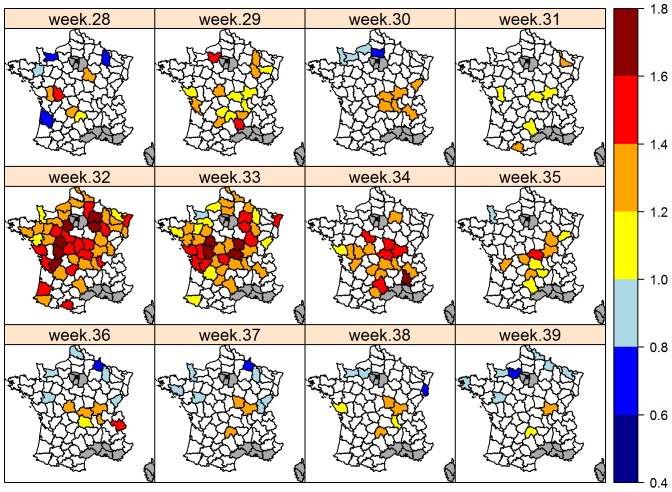
Geographic distribution of the weekly mortality ratio (O/E) in 2003. French departments in white correspond to departments where Observed/Expected mortality is not significantly different from 1. Mortality was not modeled in departments in gray due to the scarcity of cattle populations.

Excess mortality was found in 60% (48/81) and 51% (41/81) of French departments during weeks 32 and 33 respectively, and in 22% (18/81) and 12% (10/81) of departments in the two weeks following the heat wave. Departments with the highest excess mortality (SMR≥1.4) were mostly located in the center of France (Figure 4), where the heat wave lasted the longest (Figure S1). In contrast, departments with no or low excess mortality (1.0 ≤ SMR ≤ 1.2) were mostly in mountainous regions.

### Impact of the 2006 heat wave on cattle mortality

#### National analysis

The overall excess mortality of 3,601 cattle estimated in week 29 corresponds to a relative excess of 21.6% (Figure 1). Although the heat wave lasted from weeks 28 to 30, the first week went completely unnoticed (−1.4%) and excess mortality during the third week (13.9%) and the following weeks was not significant. In all, over the heat wave period (weeks 28–30), 6,193 extra deaths were estimated, corresponding to relative excess mortality of 12% [11]–[14].

All cattle subpopulations were subject to excess mortality in weeks 29 and/or 30 except beef cattle aged 2–5 years (Table 2). No excess mortality was found in any subpopulation in week 28 or after the heat wave (Figure 3). The heat wave had a higher impact on beef calves (SMR = 1.18) than dairy calves 0 to 7 days old (1.06) and a lower impact on beef cattle (1.02) than dairy cows between 2 and 5 years (1.18) (Table 2). No significant difference was found between production types for the other age classes.

All cattle subpopulations had a lower SMR during the 2006 heat wave than the 2003 one, except beef calves 0 to 7 days old and dairy cattle between 6 months and 2 years (Table 2).

#### Departmental analysis

**Figure 5:**
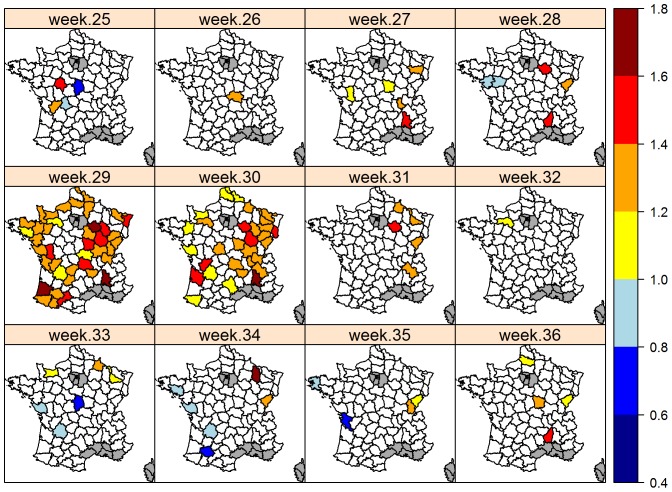
Geographic distribution of the weekly mortality ratio (O/E) in 2006. French departments in white correspond to departments where Observed/Expected mortality is not significantly different from 1. Mortality was not modeled in departments in gray due to the scarcity of cattle populations.

Excess mortality was found in three French departments (4%) in week 28, 40 departments (49%) in week 29 and 31 departments (38%) in week 30 (Figure 5). In week 31 (i.e. after the heat wave), 6% of departments suffered from significant excess mortality. Overall, the distribution of mortality reflects the spatial distribution of the heat wave, with SMR≥1.4 in southern and eastern regions (Figure 5 and Figure S1). However, departments along the north-west Atlantic coast had an SMR around 1.4, despite a limited number of hot days.

## Discussion

While multiple studies have investigated the public health impact of the heat waves that struck France in 2003 and 2006, to our knowledge this is the first study to investigate and quantify the influence of these extreme events on cattle mortality. Routinely collected data were modeled to evaluate the impact of the 2003 and 2006 heat waves on cattle mortality in France. Predictions from the different models converged to show that the 2003 heat wave had a greater impact than the 2006 one (excess mortality of 24% and 12% respectively). This excess mortality was observed during the two weeks of the 2003 heat wave and during the following weeks in certain cattle subpopulations. In contrast, excess mortality in 2006 was found during the second and third week of the heat wave only. All cattle subpopulations suffered from the heat, but there was no marked difference in excess mortality between types of production or age classes. Results by department indicated a widespread and longer impact in 2003, yet the magnitude of mortality varied among departments.

### Models

While it is common practice to make predictions for the future on the basis of retrospective time series analysis, it is unusual to make estimations for a period directly preceding the time window used to calibrate the model. A specific issue of this approach is that the model is adjusted using data including information from the population which survived the heat wave of 2003. The heat wave could have altered the mortality pattern in 2004–2005 by modifying the population's structure, and thus biased the model's calibration and possibly its predictions. However, a comparison of the age and production type distributions in the study population in 2003 versus 2004 and 2005 revealed no difference in the population structure (results not shown). Furthermore, the good fit of the national models for 2003 indicates that the mortality pattern was unchanged.

The validity of this method for estimating excess mortality relies on the model's adjustment to mortality data. The results showed that the predicted seasonal variations in mortality matched the observed mortality values well in both 2003 and 2006 for periods outside the heat waves, indicating that the marked excess mortality during the heat wave periods resulted from the extreme weather conditions. This good match is especially true for the national general model and to a lesser extent for the national categorical model. For some cattle subpopulations, unusual, short-term variations in mortality were detected. Contagious diseases, for example, may lead to a sudden increase in mortality [14]. However, no large-scale outbreaks were recorded during the study period. The anomalies observed are likely to have resulted from unforeseen events such as an increase in the culling rate due to a change in market price or unusual weather conditions (cold spells). Furthermore, the shortness of the calibration period prevented the use of trends in the models, which may explain the overestimated mortality rates in both dairy and beef calves aged from 0 to 7 days over several weeks in 2003 [19].

### Differences according to production type and age

Cattle have a remarkable ability to adjust to changes in the environment, and in particular to increasing temperatures. Early homeostatic mechanisms include increased respiration rate, reduced feed intake and lower milk production [10]. Such thermoregulatory behavior may help cattle to adjust to heat stress within certain limits, but during a heat wave, the rapid increase in temperature, prolonged thermal challenge and reduced ability to dissipate heat during the night impair physiological functions and may ultimately cause death [8], [20]. The response of cattle to stressful weather conditions depends on individual factors that include production type, age, and health [11], [20]. Dairy cattle are therefore expected to be less able to adjust to heat stress due to their higher metabolism related to milk production. Indeed, while beef cattle over two years old were not impacted by the 2006 heat wave, dairy cattle suffered a high death toll. However, during the severe heat wave of summer 2003, both types of production suffered excess mortality, indicating that beef cattle were less able to acclimatize to the extreme temperatures in 2003 than 2006. Furthermore, the results for 2003 indicated that the heat wave had a smaller impact on calves aged from 0 to 7 days than animals in other age classes, with the exception of dairy cattle between 6 months and 2 years old. This finding suggests that calves are better able to acclimatize and recover from the heat stress in 2003, undoubtedly because optimal performance and wellbeing in newborn and young calves up to one month old occur at higher temperatures than in older cattle [20]. The lower intensity of the 2006 heat wave may explain the improved ability of all cattle subpopulations to cope with the heat. Quantitative assessments of the relationship between heat exposure and animal response in given locations would clarify the effects on animal performance of the climate and other local environmental factors, like farm management, differences in stabling, adequacy of feed and water or preventive and mitigation measures.

### Comparison between 2003 and 2006

The overall excess mortality associated with the heat wave was found to be twice as high in 2003 (24%) as 2006 (12%). In addition, a two-week excess mortality period was detected in most French departments during the 2003 heat wave, whereas in 2006, only a few departments showed excess mortality for longer than one week. Although the 2006 heat wave was slightly longer (19 days, versus 15 days in 2003) and occurred earlier in the summer (which limits the population's ability to acclimatize to the heat [21]), these patterns probably reflect the higher intensity of the 2003 heat wave, with temperatures greatly exceeding those recorded in 2006 (Figure S1). Results showed that generally speaking, the modeled excess mortality distribution matched the observed spatial distribution of hot days (Figure S1). However, in some departments, SMR estimates did not reflect the intensity of the heat waves, as deduced from the number of successive hot days. Yet this indicator only provides a partial picture of the local intensity of the heat wave, because it does not take into account the gap between mean seasonal temperatures and extreme temperatures recorded during the heat wave. Thus, in departments with relatively low seasonal temperatures (e.g. along the north-west Atlantic coast), cattle are likely to be more affected by the heat stress and consequently suffer a higher death toll than herds in departments which regularly have hot summer temperatures (southern France). Further analyses are needed to evaluate the relationship between exposure and mortality, and identify the causes of the excess mortality revealed one or more weeks after the heat wave in 2003 but not in 2006.

### Preventive measures

Global warming is predicted to increase the frequency of heat waves as well as global mean temperatures [22], [23], increasing health risks. The increased awareness of the risk for humans after the 2003 heat wave led to the implementation of preventive measures that reduced excess mortality in 2006 by two-thirds [7] and reduced the impact of the moderate 2010, 2011 and 2012 heat waves [13]. The results of the present study underline the negative impacts of the 2003 and 2006 heat waves on cattle mortality and previous veterinary studies have already demonstrated the potential indirect economic effects due to lower reproductive efficiency and animal production [11]. These findings underline the need to use preventive management practices to reduce environmental challenges to cattle and other farm animals [8]–[11]. Specific measures might include a climate-based early warning system to alert farmers and veterinarians of the impeding danger, and practical solutions to reduce livestock vulnerability to heat stress, such as indoor cooling systems, provision of shade in pastures and a ban on transport during the heat of the day.

## Supporting Information

Figure S1 Number of days with maximal temperatures above 35°C during the summer heat waves of 2003 (August 2 to 14) and 2006 (July 10 to 28) in France. These maps were obtained from the French national meteorological service.
(TIFF)
