# Supplementary figures and images for: Assessment of the Impact of the 2003 and 2006 Heat Waves on Cattle Mortality in France
Source: PLoS One. 2014 Mar 25;9(3):e93176. doi: 10.1371/journal.pone.0093176 (PMC3965539; doi:10.1371/journal.pone.0093176)

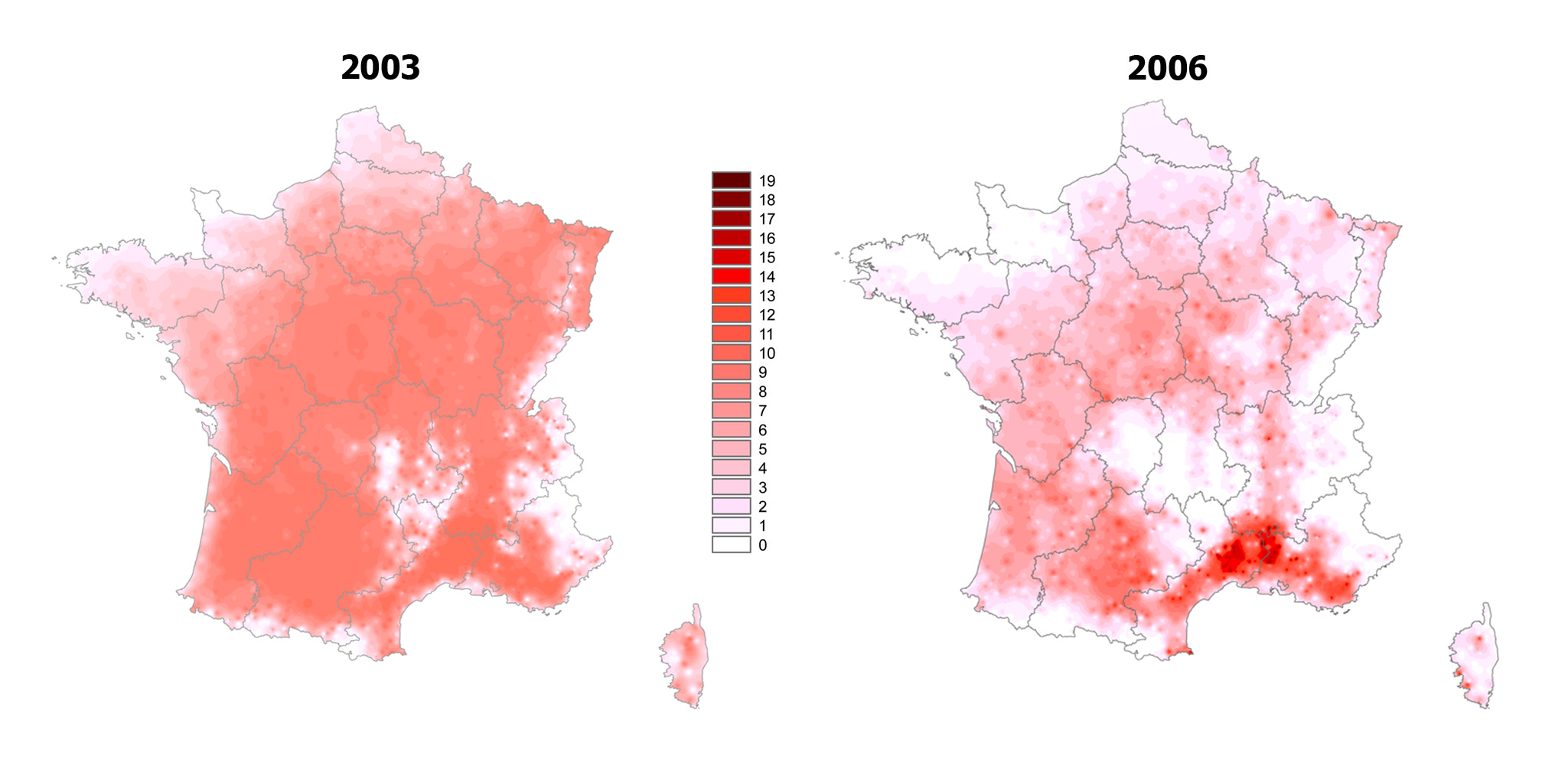

Supplement: Figure S1 — Number of days with maximal temperatures above 35°C during the summer heat waves of 2003 (August 2 to 14) and 2006 (July 10 to 28) in France. These maps were obtained from the French national meteorological service. (TIFF) [file pone.0093176.s001.tiff]
